# Fachärztliche Unterversorgung bei Heimbewohnern – Prävalenzstudie und Hochrechnung

**DOI:** 10.1007/s00391-021-01865-z

**Published:** 2021-03-16

**Authors:** Maike Schulz, Jonas Czwikla, Annika Schmidt, Chrysanthi Tsiasioti, Antje Schwinger, Ansgar Gerhardus, Guido Schmiemann, Karin Wolf‑Ostermann, Heinz Rothgang

**Affiliations:** 1 SOCIUM – Forschungszentrum Ungleichheit und Sozialpolitik, Universität Bremen, Mary-Somerville-Straße 5, 28359 Bremen, Deutschland; 2 Institut für Public Health und Pflegeforschung (IPP), Universität Bremen, Grazer Straße 4, 28359 Bremen, Deutschland; 3 Wissenschaftliches Institut der AOK (WIdO), Postfach 11 02 46, 10832 Berlin, Deutschland; 4 Wissenschaftsschwerpunkt Gesundheitswissenschaften, Universität Bremen, Bremen, Deutschland

**Keywords:** Stationäre Pflegeeinrichtung, Arztkontakte, Chronische Erkrankungen, Multimorbidität, Routinedaten, Resident nursing home, Medical contact, Chronic diseases, Multimorbidity, Routine data

## Abstract

**Hintergrund:**

Bisherige Studien deuten darauf hin, dass Pflegebedürftige eine geringere fachärztliche Versorgung aufweisen als Nichtpflegebedürftige. Insbesondere im stationären Setting ist die fachärztliche Versorgungsintensität gering. Aus den bestehenden quantitativen Versorgungsunterschieden lässt sich bislang jedoch nicht ableiten, inwieweit von einer Unterversorgung bei Pflegebedürftigen ausgegangen werden muss. Für die Versorgungsbereiche Sehfähigkeit, Hörfähigkeit, Mundgesundheit und Parkinson-Syndrom wird geprüft, inwieweit Heimbewohner fachärztlich unterversorgt sind.

**Material und Methoden:**

In 44 Pflegeheimen in Bremen und Niedersachsen wurde der Gesundheitszustand von 409 Pflegebedürftigen mittels standardisierter Assessments und Befragungen erhoben; zusätzlich wurden Diagnosen und die medizinische Versorgung aus der Pflegedokumentation ausgewertet. Ärzteteams beurteilten auf dieser Grundlage für jeden Pflegebedürftigen, inwieweit eine bedarfsgerechte fachärztliche Versorgung vorlag oder nicht.

**Ergebnisse:**

Gemäß ärztlichem Urteil zeigt sich bei 45 % (Sehfähigkeit), 19 % (Parkinson-Syndrom), 16 % (Mundgesundheit) und 15 % (Hörfähigkeit) der Bewohner mit entsprechendem Versorgungsbedarf eine fachärztliche Unterversorgung. Bei 27 % aller Bewohner zeigt sich in mindestens einem der 4 Versorgungsbereiche eine fachärztliche Unterversorgung. Hochgerechnet entspricht dies bis zu 205.000 fachärztlich unterversorgten Pflegeheimbewohnern in Deutschland.

**Diskussion:**

Die Studie liefert für ausgewählte Versorgungsbereiche die ersten empirischen Belege über das Ausmaß fachärztlicher Unterversorgung von Pflegeheimbewohnern. Daher erscheint es notwendig, entsprechende Interventionen zur Reduktion der Unterversorgung zu entwickeln und zu erproben.

**Zusatzmaterial online:**

Die Online-Version dieses Beitrags (10.1007/s00391-021-01865-z) enthält eine detaillierte Erklärung zur Methodik der Datenerhebung sowie 6 Tabellen mit weiterführenden Analysen. Beitrag und Zusatzmaterial stehen Ihnen im elektronischen Volltextarchiv auf https://www.springermedizin.de/link/10.1007/10.1007/s0391-021-01865-z zur Verfügung. Sie finden das Video am Beitragsende unter „Supplementary Material“.

## Abstract (en)

**Background:**

Nursing home residents tend to have lower medical specialist utilization than other groups of older people; however, as yet there is little evidence whether nursing home residents obtain adequate medical specialist care. This study investigated whether nursing home residents receive adequate oral health care, ophthalmological care, otorhinolaryngologist care and neurological care. The unmet needs of the nursing home population in Germany was extrapolated.

**Material and methods:**

Audiometry, eye examinations and oral visual inspection were performed in 409 residents from 44 nursing homes. Medical care in the previous 12 months as well as existing diagnoses were retrieved from the nursing documentation. Teams of physicians evaluated for each resident based on all collected data if the resident obtained specialist care that was adequate to the needs.

**Results:**

Between 15% and 45% of the residents with need for medical specialist care did not receive adequate specialist care. Of all residents 27% had unmet need of specialist care in at least one of the investigated medical specialties. It is projected that up to 205,000 nursing home residents in Germany do not receive adequate medical specialist care.

**Conclusion:**

Given a considerable proportion of nursing home residents with unmet need of specialist care, interventions should be developed that help reduce the level of unmet needs.

## Hintergrund

Die medizinische Versorgung in Pflegeeinrichtungen stellt für alle Beteiligten eine große Herausforderung dar. Pflegeheimbewohner weisen eine höhere Morbidität auf und haben dadurch einen höheren Bedarf an medizinischer Versorgung als ambulant gepflegte Pflegebedürftige und Nichtpflegebedürftige [1, 2]. Gleichzeitig ist die medizinische Versorgung bei Pflegebedürftigen schwieriger zu bewerkstelligen, weil viele Pflegebedürftigen demenziell erkrankt [3] und/oder in ihrer Mobilität eingeschränkt sind [4]. Dies erschwert Untersuchungen, den Gang in die Arztpraxis und die Kommunikation zwischen Arzt und Patient. Hinzu kommt bei Pflegeheimbewohnern, dass die Angehörigen oftmals weniger unterstützend in die medizinische Versorgung involviert sind, als es bei Pflegebedürftigen in der eigenen Häuslichkeit der Fall ist [5]. Auch die Zusammenarbeit zwischen Ärzten und Pflegepersonal wird als verbesserungswürdig angesehen [6].

In der Konsequenz scheinen Heimbewohner im Vergleich zu ambulant gepflegten Pflegebedürftigen und Nichtpflegebedürftigen insbesondere hinsichtlich der fachärztlichen Versorgung in geringerem Umfang versorgt zu sein. Nur wenige Fachärzte machen regelmäßige Visiten im Pflegeheim [7]. Betrachtet man die Studienlage, zeigt sich bei Heimbewohnern eine geringere Inanspruchnahme von Fachärzten nahezu aller Fachrichtungen [8]: In der Tendenz haben sie weniger Internisten‑, Augenarzt- und Hals-Nasen-Ohren(HNO)-Arzt-Kontakte als Nichtpflegebedürftige; auch bei Urologen und Gynäkologen ist die Kontakthäufigkeit vergleichsweise niedrig [9–11]. Nur ein Bruchteil der Heimbewohner hat mindestens einen Zahnarztkontakt pro Jahr [12]. Zwar weisen Pflegebedürftige mehr Hausarztkontakte auf als Nichtpflegebedürftige [8]; ob diese allerdings fachärztliche Kontakte ersetzen können, ist fraglich. Lediglich hinsichtlich der neurologischen Versorgung zeigt sich bei Pflegebedürftigen eine höhere Inanspruchnahme, selbst wenn für die Tatsache kontrolliert wird, dass Pflegebedürftige mehr neurologische Erkrankungen aufweisen als Nichtpflegebedürftige [13]. Darüber hinaus konnte gezeigt werden, dass es mit höherer Pflegestufe umso wahrscheinlicher ist, bei einer Erkrankung keinen entsprechenden Facharztkontakt zu haben [14].

Zwar ist damit eine geringere Versorgung der Heimbewohner belegt, es kann daraus aber nicht zwingend auf eine Unterversorgung geschlussfolgert werden. Denn die empirisch beobachtete geringere fachärztliche Inanspruchnahme ist nicht gleichzusetzen mit Unterversorgung. Denn zum einen wurden bestehende Morbiditätsunterschiede zwischen Pflegebedürftigen und Nichtpflegebedürftigen in Studien zum Inanspruchnahmeverhalten bislang nicht ausreichend kontrolliert, weil in der Regel nur begrenzt Informationen zur Morbidität vorhanden sind [10, 11]. Zum anderen fehlt es derlei empirischen Auswertungen an einem Bewertungsmaßstab, um zu beurteilen, ob Pflegebedürftige unterversorgt oder Nichtpflegebedürftige vielmehr überversorgt sind.

Das Ziel der vorliegenden Studie war es daher, für 4 Versorgungsbereiche auf Grundlage ärztlicher Beurteilung und unter Berücksichtigung des Allgemeinzustands der Heimbewohner zu prüfen, inwieweit die Bewohner tatsächlich fachärztlich unterversorgt sind. Die Definition bedarfsgerechter Versorgung orientiert sich hierbei an den Überlegungen des Sachverständigenrats im Gesundheitswesen [15] und liegt vor, wenn (a) fachärztliche Versorgung bei einem bestehenden und behandelbaren (subjektiven oder objektiven) Gesundheitsproblem erfolgt ist oder (b) keine fachärztliche Versorgung erfolgt ist, wenn kein Gesundheitsproblem besteht oder wenn ein Gesundheitsproblem nicht oder nur ohne gesundheitlichen Nettonutzen behandelbar ist. Von fachärztlicher Unterversorgung wird im Umkehrschluss ausgegangen, wenn bei einem bestehenden und behandelbaren subjektiven oder objektiven Gesundheitsproblem keine fachärztliche Versorgung erfolgt ist.

## Methodik

Es handelt sich um eine Querschnittsstudie, die Teil des Projekts „Bedarfsgerechtigkeit der medizinischen Versorgung Pflegebedürftiger in stationären Einrichtungen (MVP-STAT)“ ist [16]. Das Projekt wurde mit Mitteln des Innovationsausschusses beim Gemeinsamen Bundesausschuss unter dem Förderkennzeichen 01VSF16039 gefördert. Teilergebnisse des Projekts wurden bereits veröffentlicht [13, 14]; ein Gesamtbericht des Projekts mit weiteren Analysen auf Basis dieser Daten wird nach Ende des Projekts durch den Mittelgeber veröffentlicht.

Die Stichprobengewinnung erfolgte von Januar bis Dezember 2018 in stationären Pflegeeinrichtungen im Bundesland Bremen bei Bewohnern, die bei der Allgemeinen Ortskrankenkasse (AOK) Bremen/Bremerhaven oder Niedersachsen versichert waren. Ab April 2018 wurde die Stichprobengewinnung auf alle anderen Versicherten und schrittweise auch auf das umliegende Niedersachsen ausgeweitet, um die angestrebte Fallzahl zu erreichen. Eine Erweiterung der Stichprobe wurde bereits als Option im Studienprotokoll dokumentiert. Die angestrebte Stichprobengröße betrug 500. Es handelt sich um eine Gelegenheitsstichprobe.

Bei der Stichprobengewinnung wurden zuerst die Pflegeheimleitungen zur Teilnahme eingeladen; bei Einwilligung wurden in einem zweiten Schritt die einschlussfähigen Bewohner zur Teilnahme eingeladen. Einschlusskriterien für die Teilnahme waren, dass die Bewohner zum Zeitpunkt der Datenerhebung bereits 12 Monate in der Pflegeeinrichtung lebten und mindestens 60 Jahre alt waren. Detaillierte Informationen zu den erhobenen und ausgewerteten Daten werden im Zusatzmaterial online gegeben.

### Beurteilung der Bedarfsgerechtigkeit der fachärztlichen Versorgung

Sechs Ärzte (Fachärzte für Allgemeinmedizin und internistische Medizin, teilweise mit geriatrischer Weiterbildung) bildeten wechselnde Zweierteams und beurteilten zwischen Juni 2018 und Mai 2019 für jeden Bewohner, ob die individuelle fachärztliche Versorgung in den vorangegangenen 12 Monaten bedarfsgerecht war oder nicht.

Die Ärzte wurden wie folgt angeleitet: Sie beurteilten, inwieweit zum Erhebungszeitpunkt objektiver und/oder subjektiver Bedarf vorlagen/vorlag, und ob durch fachärztliche Versorgung ein Nutzen zu erreichen sei, der die Risiken und Nachteile eines Kontakts überwiege. Dabei wurden auch leitlinienbasierte, empfohlene Kontrolluntersuchungen berücksichtigt. Zur Beurteilung standen den Ärzten alle Ergebnisse der Assessments, Selbst- und der Fremdeinschätzung zur Verfügung, inklusive Interpretationshilfen; außerdem die Informationen zu Diagnosen, Medikation und zur medizinischen Versorgung aus der Pflegedokumentation.

Im Abgleich mit dem Bedarf beurteilten die Ärzte, ob in den vorangegangenen 12 Monaten fachärztliche Kontakte in einem bedarfsgerechten Ausmaß erfolgten. Wenn die Anzahl der Kontakte nicht als bedarfsgerecht beurteilt wurde, wurde entschieden, ob eine fachfachärztliche Unterversorgung oder Überversorgung vorlag.

Die Ärzte konnten die Versorgung als „bedarfsgerecht“, „unterversorgt“, „überversorgt“ oder „nichtbeurteilbar“ beurteilen und begründeten ihr Urteil schriftlich. Um zu gewährleisten, dass die fachärztliche Versorgung der Bewohner von allen ärztlichen Teams in vergleichbarer Weise beurteilt wird, wurde ein Teil der Fälle doppelt, d. h. jeweils unabhängig von 2 ärztlichen Teams, beurteilt und abgeglichen.

## Ergebnisse

Insgesamt wurden 363 stationäre Pflegeeinrichtungen in Bremen und Niedersachsen schriftlich und telefonisch zur Teilnahme eingeladen. Die Datenerhebung wurde in 44 stationären Pflegeeinrichtungen durchgeführt; dies entspricht einer Teilnahmequote von 12 %. Aus den 44 Pflegeheimen wurden von 518 Bewohnern bzw. deren rechtlichen Betreuern Teilnahmeeinwilligungen eingesendet. Bei 76 Bewohnern konnte die Datenerhebung nicht durchgeführt werden, weil diese in der Zeit zwischen Teilnahmeeinwilligung und Datenerhebung verstorben waren oder weil sie am Tag der Erhebung ihre Teilnahmeeinwilligung zurückzogen. Die Datenerhebung konnte folglich bei 442 Bewohnern durchgeführt werden. Von den 442 Bewohnern wurden 33 von der Analyse ausgeschlossen, da sie die Einschlusskriterien nicht erfüllten. Dies ergibt 409 gültige Fälle. Diese teilen sich auf in 273 Frauen (67 %) und 136 Männer (33 %). Das Alter zum Erhebungszeitpunkt reichte von 60 bis 108 Jahren (Zusatzmaterial online: Tab. 1 und 2).

### Gesundheitszustand und (fach)ärztliche Versorgung

Die Assessments konnten bei 62 % (Mundinspektion), 55 % (Hörtest) und 54 % (Sehtest binokular) der Pflegeheimbewohner durchgeführt werden. Die Hälfte der Bewohner, die an den Seh- und Hörtests teilnahmen, bestanden diese nicht. 55 % der Bewohner, bei denen eine Mundinspektion durchgeführt wurde, wiesen einen auffälligen Mundzustand auf.

Subjektiv unzufrieden hinsichtlich ihrer Sehfähigkeit waren 10 % der Bewohner; 6 % waren mit ihrer Hörfähigkeit nicht zufrieden, und Beschwerden bezüglich der Mundgesundheit gaben 28 % der Bewohner an. Die Bezugspflegekräfte gaben eine Fremdeinschätzung zum Gesundheitszustand der Bewohner an, aus der sich ableiten ließ, dass 11 % der Bewohner aufgrund mangelnder Hörfähigkeit nicht telefonieren könnten, 23 % der Bewohner Kleingedrucktes nicht lesen könnten und 4 % aller Bewohner zahnlos seien (Zusatzmaterial online: Tab. 3).

In den vergangenen 12 Monaten hatten 98 % der Bewohner mindestens einen hausärztlichen Kontakt irgendeiner Art (Pflegeheimvisite, Praxisbesuch, oder telefonischer Kontakt). Dagegen hatte im gleichen Zeitraum nur rund ein Fünftel der Bewohner überhaupt einen fachärztlichen Kontakt irgendeiner Art (Zusatzmaterial online: Tab. 4).

### Beurteilung der fachärztlichen Versorgung

Die ärztlichen Fallbewertungen ergeben auf Grundlage des Allgemein- und Gesundheitszustandes (Zusatzmaterial online: Tab. 3) und der medizinischen Versorgung (Zusatzmaterial online: Tab. 4) der Bewohner, dass sich bei mehr als einem Viertel (27 %) aller Bewohner in mindestens einem der untersuchten Versorgungsbereiche eine fachärztliche Unterversorgung zeigt. Je nach Versorgungsbereich weisen zwischen 15 und 45 % der Bewohner mit Versorgungsbedarf eine fachärztliche Unterversorgung auf (Tab. 1). Der höchste Anteil an Unterversorgung besteht demgemäß im Versorgungsbereich Sehfähigkeit; hier ist fast die Hälfte (45 %) der Bewohner mit Versorgungsbedarf fachärztlich unterversorgt. In den anderen Versorgungsbereichen liegt der Anteil der Unterversorgten bei 19 % (Parkinson-Syndrom), 16 % (Mundgesundheit) und 15 % (Hörfähigkeit). Überversorgung lag bei 5 Personen in jeweils einem Versorgungsbereich vor. Bei 16 Bewohnern konnte die fachärztliche Versorgung in einem oder mehreren Versorgungsbereichen aufgrund widersprüchlicher oder fehlender Informationen nicht beurteilt werden. Über alle 4 Versorgungsbereiche hinweg weisen 111 der 409 Bewohner in mindestens einem Versorgungsbereich eine Unterversorgung auf; dies entspricht einem Anteil von 27 %.

**Table 1:** 

	Stichprobe Pflegeheimbewohner			Hochrechnung Pflegeheimbewohner in Bremen und Niedersachsen			Hochrechnung Pflegeheimbewohner in Deutschland		
	Gesamt^a^	Davon mit Versorgungsbedarf	Davon unterversorgt (95 %-Konfidenzintervall)	Gesamt	Davon mit Versorgungsbedarf	Davon unterversorgt (%)	Gesamt	Davon mit Versorgungsbedarf	Davon unterversorgt (%)
*Sehfähigkeit*	400	115	52 (45 %; 40–50 %)	93.186	26.790	12.544 (47 %)	755.631	217.243	101.681 (47 %)
*Hörfähigkeit*	400	101	15 (15 %; 12–19 %)	93.186	23.529	3575 (15 %)	755.631	190.797	28.981 (16 %)
*Mundgesundheit*	401	401	64 (16 %; 12–20 %)	93.186	93.186	14.416 (15 %)	755.631	755.631	116.973 (15 %)
*Parkinson-Syndrom*	31	31	6 (19 %; 5–33 %)	7063	7063	1309 (19 %)	57.273	57.273	10.460 (19 %)

Die Berechnung der 95 %-Konfidenzintervalle basiert auf der Größe der Stichprobe (Spalte „Gesamt“) und dem Anteil der Unterversorgten in der Stichprobe als Schätzer für die Grundgesamtheit. Die Hochrechnung erfolgt auf gesetzlich und privat versicherte Pflegeheimbewohner im Alter über 60 Jahre, basierend auf Daten der Pflegestatistik 2017

^a^Diese Spalte beinhaltet alle Fälle, die als bedarfsgerecht oder unterversorgt beurteilt wurden

Zur Überprüfung der Interrater-Reliabilität wurden 48 der 409 Fälle ärztlich doppelt bewertet. Es zeigt sich generell eine hohe Übereinstimmung in den Bewertungen; Zwischen 72,9 % (Versorgungsbereich Mundgesundheit) und 95,8 % (Versorgungsbereich Parkinson-Syndrom) der Fälle wurden identisch bewertet.

### Nonresponderanalyse und Hochrechnung

Die Ergebnisse der Nonresponderanalyse deuten an, dass von den 7 soziodemografischen bzw. gesundheitsbezogenen Merkmalen 3 mit der Teilnahmewahrscheinlichkeit signifikant korrelieren: Jüngere Bewohner sind unter den Studienteilnehmern signifikant überrepräsentiert. Die Bewohner in der Stichprobe zeichnen sich außerdem im Vergleich zu den Bewohnern, die nicht an der Studie teilgenommen haben, durch eine niedrigere Anzahl von hausärztlichen Kontakten aus. Darüber hinaus sind Bewohner unterrepräsentiert, die noch im Erhebungsjahr verstorben sind.

Bei der Hochrechnung kann von diesen 3 Merkmalen, die im Rahmen der Nonresponderanalyse identifiziert wurden, nur eine Anpassung an die Altersstruktur der Grundgesamtheit vorgenommen werden, da die Pflegestatistik des Statistischen Bundesamtes die Zahl der Heimbewohner nicht nach Mortalität oder Hausarztkontakten aufschlüsselt.

Die Altersadjustierung im Rahmen der Hochrechnung erfolgte wie folgt: Für die Stichprobe wurde der Anteil der unterversorgten Bewohner mit Versorgungsbedarf getrennt nach Altersgruppen (60–64, 65–69, 70–74, 75–79, 80–84, 85–89, 90+) berechnet. Der resultierende Stichprobenanteilswert je Altersgruppe wurde mit dem entsprechenden Altersgruppenanteil der Heimbewohner in Bremen und Niedersachsen gemäß Pflegestatistik gewichtet. Daraus resultiert die Schätzung des hochgerechneten und altersadjustierten Anteils der fachärztlich unterversorgten Bewohner in Bremen, Niedersachsen und Deutschland. Die Hochrechnung erfolgt auf gesetzlich und privat versicherte Pflegeheimbewohner im Alter über 60 Jahre, basierend auf Daten der Pflegestatistik 2017. Wie sich zeigt, verändert diese Anpassung an die Altersstruktur die Anteilswerte der fachärztlich Unterversorgten nur geringfügig (Tab. 1).

Die Hochrechnung auf die Heimbewohner in Bremen und Niedersachsen ergibt in absoluten Zahlen, dass von den insgesamt rund 93.000 Heimbewohnern in Bremen und Niedersachsen bis zu ca. 14.400 (Versorgungsbereich Mundgesundheit) fachärztlich unterversorgt sind. Hochgerechnet auf die rund 756.000 Pflegeheimbewohner in Deutschland (Stichtag 31.12.2017) entspricht dies bis zu 117.000 (Versorgungsbereich Mundgesundheit) fachärztlich unterversorgten Heimbewohnern.

## Diskussion

Die vorliegende Studie liefert erste Befunde zum Ausmaß fachärztlicher Unterversorgung von Pflegeheimbewohnern in Deutschland. Mehr als ein Viertel der Heimbewohner mit Versorgungsbedarf sind in mindestens einem der 4 untersuchten Versorgungsbereiche fachärztlich unterversorgt – in absoluten Zahlen entspricht dies rund 205.000 Heimbewohnern in Deutschland. Je nach Versorgungsbereich sind zwischen 15 und 47 % der Bewohner mit Versorgungsbedarf fachärztlich unterversorgt. Insbesondere bei der augenärztlichen Versorgung ist der Anteil der Unterversorgten auffallend hoch.

Alleinstellungsmerkmal dieser Studie ist die umfassende Erhebung von Daten zum Gesundheitszustand, die in bisherigen Sekundärdatenanalysen nicht vorlagen. Eine Stärke unseres Vorgehens ist außerdem, dass wir auch die große Gruppe der demenziell erkrankten Heimbewohner in die Studie einschließen konnten, indem deren Gesundheitszustand über eine Fremdeinschätzung erfasst wurde.

Vergleichbare Aussagen zum Ausmaß von Unterversorgung in der fachärztlichen Versorgung von Pflegebedürftigen liegen bislang nur vereinzelt vor. Häufig adressieren empirische Studien zur medizinischen Versorgung von Heimbewohnern deren Arzneimittelversorgung und konstatieren diesbezüglich Defizite [17, 18]. Die Anzahl der haus- und fachärztlichen Arztkontakte wurde ebenfalls häufig untersucht, da es Heimbewohnern vergleichsweise schwerfällt, Arzttermine zu koordinieren und eine Arztpraxis zu aufzusuchen. Unsere Ergebnisse deuten an, dass fachärztliche Kontakte nur bei einem Fünftel der Bewohner stattfinden. Dieser Befund bestätigt damit die Ergebnisse vorheriger Studien; lediglich für zahnärztliche Kontakte liegt die Kontaktwahrscheinlichkeit in der Regel höher [19, 20]. Auch zeigen sich in Analysen auf Basis von Sekundärdaten tendenziell mehr fachärztliche Kontakte als in den hier analysierten Primärdaten [11].

Nur wenige bestehende Studien zur Inanspruchnahme von Arztkontakten können allerdings Aussagen darüber machen, inwieweit der Umfang der Inanspruchnahme von Pflegeheimbewohnern bedarfsgerecht ist. Im Hinblick auf die augenärztliche Versorgung wurde in bisherigen Studien angenommen, dass 20–40 % der Pflegebedürftigen einen unbehandelten Versorgungsbedarf oder fehlende Kontrolluntersuchungen aufweisen [21–23]. Wir kommen hierbei sogar zu noch höheren Werten. Zu den Versorgungsbereichen Hörfähigkeit, Mundgesundheit und Parkinson-Syndrom liegen bislang keine spezifischen Erkenntnisse zur fachärztlichen Unterversorgung vor.

Die Berücksichtigung der subjektiven Wahrnehmung der Bewohner hinsichtlich ihrer gesundheitlichen Fähigkeiten zusätzlich zu den objektiven Gesundheitsassessments bietet eine realistischere Einschätzung der Bedarfsgerechtigkeit der Versorgung. Es zeigte sich allerdings, dass die Mehrheit trotz objektiver Einschränkungen mit dem eigenen Gesundheitszustand subjektiv zufrieden ist. Eine solche Diskrepanz zwischen objektiven und subjektiven Indikatoren des Gesundheitszustandes zeigte sich bereits in anderen Studien mit Pflegebedürftigen [12, 24]; die gesundheitlichen Beeinträchtigungen werden offenbar subjektiv nicht als solche wahrgenommen. Dies wurde bei der Beurteilung der fachärztlichen Versorgung berücksichtigt und führt zu einem geringeren Anteil fachärztlicher Unterversorgung als es bei einer Bewertung auf Basis rein objektiver Faktoren der Fall wäre.

Unsere Methodik weist jedoch auch Limitationen auf: Die Repräsentativität der Ergebnisse wird durch die Tatsache einschränkt, dass es sich um eine Gelegenheitsstichprobe handelt. Zwar deutet die Nonresponderanalyse an, dass nur geringfügige Merkmalsunterschiede zwischen teilnehmenden und nichtteilnehmenden Bewohnern bestehen; diese ist allerdings auf AOK-Versicherte beschränkt, weil nur für diese Versicherten entsprechende Daten zur Verfügung standen.

Bedarfsgerechte fachärztliche Versorgung wird in dieser Studie über die Anzahl der fachärztlichen Kontakte definiert. Dies lenkt den Fokus auf den Zugang zur fachärztlichen Versorgung und deren Umfang, blendet die Qualität der fachärztlichen Behandlung hingegen aus. Das Konzept „Versorgungsbedarf“ wurde für die 4 Versorgungsbereiche nicht konsistent gestaltet; dies lag darin begründet, dass widersprüchliche Zuordnungen vermieden werden sollten (z. B. die Zuweisung „kein Versorgungsbedarf“ in Kombination mit dem ärztlichen Urteil „fachärztlich unterversorgt“). Dies erschwert den Vergleich der Ergebnisse in den 4 Versorgungsbereichen. Eine Definition von Versorgungsbedarf, die ausschließlich auf einem klar eingrenzbaren Gesundheitsproblem beruht, bietet eine bessere Vergleichbarkeit innerhalb der Studie und auch darüber hinaus mit anderen Studien.

Des Weiteren wurden für die Hochrechnung Annahmen getroffen, die aufgrund fehlender Daten nicht überprüft werden können: Zum einen wird angenommen, dass die Merkmalsunterschiede, die in der Nonresponderanalyse zwischen teilnehmenden und nichtteilnehmenden Heimbewohnern der AOK identifiziert wurden, in der Grundgesamtheit bei Pflegeheimbewohnern anderer Krankenkassen ähnlich ausfallen [25]. Zum anderen wird angenommen, dass das Ausmaß des fachärztlichen Versorgungsbedarfs, den wir in der Stichprobe identifiziert haben, in der Grundgesamtheit vergleichbar ausfällt. Zudem wird angenommen, dass die Heimbewohner in der Stichprobe hinsichtlich Krankenkassenzugehörigkeit sowie soziodemografischer Merkmale (Alter, Geschlecht, finanzielle Situation u. Ä.) vergleichbar sind mit der Grundgesamtheit.

In dieser Studie wurden nur 4 fachärztliche Versorgungsbereiche betrachtet. Es lässt sich aus diesen Ergebnissen nicht darauf schließen, inwieweit in anderen medizinischen Fachgebieten eine Unterversorgung von Heimbewohnern besteht. Denn die Ergebnisse offenbaren bereits zwischen diesen 4 Versorgungsbereichen große Unterschiede im Ausmaß der Unterversorgung. Zukünftige Studien sollten somit die Unterversorgung in anderen fachärztlichen Bereichen analysieren; denn die Folgen von fachärztlicher Unterversorgung in Versorgungsbereichen wie beispielsweise der Kardiologie können lebensbedrohlich sein.

## Fazit für die Praxis

- Ein substanzieller Anteil der Pflegeheimbewohner hat fachärztlichen Versorgungsbedarf, erhält aber keine bedarfsgerechte fachärztliche Versorgung.
- Es empfiehlt sich, die Ursachen der Unterversorgung zu identifizieren, um daraus Maßnahmen zur Reduzierung der Unterversorgung abzuleiten.

## Supplementary Information










