# Supplementary material for: Medical specialist undertreatment in nursing home residents—Prevalence and extrapolation
Source: Z Gerontol Geriatr. 2021 Mar 16;54(5):479–84. [Article in German] doi: 10.1007/s00391-021-01865-z (PMC8354900; doi:10.1007/s00391-021-01865-z)
Supplement: Supplementary file 1 [file 391_2021_1865_MOESM1_ESM.pdf]

Ethik und Datenschutz

Vor Beginn der Datenerhebung wurde eine Zusammenfassung des Studienvorhabens inklusive Datenschutzkonzept der Ethikkommission der Universität Bremen vorgelegt, die das Vorhaben positiv begutachtete.

Ein dreiseitiges Informationsschreiben wurde von den Mitarbeitern der Pflegeheime an die einschlussfähigen Bewohner und/oder deren Betreuer weitergeleitet. Wenn ein Bewohner rechtlich betreut wurde oder einen Bevollmächtigten hat, wurde das Informationsschreiben an den Betreuer bzw. Bevollmächtigten gesendet. Teilweise wurden die Informationsschreiben auch an den Bewohner und Betreuer bzw. Bevollmächtigten weitergeleitet, wenn die Betreuung nur für bestimmten Aufgabenbereiche gilt. Das Informationsschreiben enthielt Informationen zum Ziel der Studie, zu den Erhebungsinstrumenten und zur Verwendung und Schutz der erhobenen Daten. Zusätzlich enthielt das Informationsschreiben Telefon- und Emailadresse eines Projektmitarbeiters, damit die Bewohner/Betreuer offene Fragen klären konnten. Dem Informationsschreiben lag ein vorfrankierter Rückumschlag bei, mit dem die unterschriebene Teilnahmeeinwilligung zurückgesendet werden konnte.

Erhebung des Gesundheitszustands und der medizinischen Versorgung

Die Datenerhebung erfolgte von Februar 2018 bis Februar 2019. Bei der Datenerhebung wurde der Gesundheitszustand mittels standardisierter Assessments und Selbsteinschätzung der Pflegebedürftigen erhoben; zusätzlich wurde eine Fremdeinschätzung durch eine Bezugspflegekraft erfasst. Die Daten wurden von geschulten Studienassistenten erhoben, die eine abgeschlossene Ausbildung in einem Gesundheitsfachberuf aufwiesen. Die Schulung erfolgte durch eine wissenschaftliche Projektmitarbeiterin mit abgeschlossener Ausbildung in einem Gesundheitsfachberuf sowie durch einen Zahnarzt, der die Inspektion des Mundraumes schulte.

Im Hinblick auf die fokussierten Versorgungsbereiche Sehfähigkeit, Hörfähigkeit, Mundgesundheit und Parkinson-Syndrom wurden Sehtest und Hörtest durchgeführt und der Mundraum wurde inspiziert. Nah- und Fernvisus wurde mittels mobiler ETDRS-Sehtafeln mit einem Abstand von 40 cm bzw. 200 cm ermittelt. Die Auswertung erfolgte gemäß WHO-Kriterien [17]. Bewohner, die eine Brille trugen, führten den Sehtest mit Brille durch. Die Hörfähigkeit wurde für jedes Ohr mittels eines Flüstertests durchgeführt. Dabei wurden insgesamt sechs Buchstaben/Zahlen ins jeweilige Ohr geflüstert. Der Flüstertest galt als bestanden, wenn 3 der 6 Buchstaben/Zahlen korrekt wiedergegeben wurden. Konnte ein Buchstabe bzw. eine Zahl nach dem ersten Hören nicht wiedergegeben werden, wurde maximal eine Wiederholung pro Ohr durchgeführt. Der Mundgesundheitszustand wurde optisch ohne zahnärztliches Equipment auf Grundlage des Oral Health Assessment Tool (OHAT) beurteilt. Das originär englischsprachige Instrument wurde eigens übersetzt und entspricht einer inzwischen validierten Übersetzung [18].

Zusätzlich beurteilten die Bewohner ihre subjektive Seh- und Hörfähigkeit im Alltag und ihre mundgesundheitsbezogene Lebensqualität (OHIP G-14) [19]. Die Fremdeinschätzung des Gesundheitszustandes wurde für alle Bewohner durch eine Bezugspflegekraft erhoben und ist insbesondere für diejenigen Bewohner wichtig, die stark dementiell erkrankt sind.

Aus der Pflegedokumentation der pflegebedürftigen Studienteilnehmer wurden bestehende Diagnosen, Medikation, Pflegegrad und die ärztliche Versorgung extrahiert. Hinsichtlich der

haus- und fachärztlichen Versorgung wurden telefonische Kontakte, Visiten in der ärztlichen Praxis sowie Pflegeheimvisiten der letzten 12 Monate erfasst.

Außerdem wurde der Allgemeinzustand mit etablierten Instrumenten erhoben: Diese umfassten die kognitive Leistungsfähigkeit (Mini Mental Status Test (MMST), den emotionalen Zustand (Geriatrischen Depressionsskala (GDS-15) und Cornell Depressionsskala (CDS)), die alltäglichen Fähigkeiten (Erweiterte Barthel Index (EBI)). Zwei Projektmitarbeiter pilotierten alle Erhebungsinstrumente in einem Pflegeheim bei vier Bewohnern. Es erfolgte keine statistische Auswertung der Reliabilität der Erhebungsinstrumente.

<u>Festlegung des Versorgungsbedarfs und Berechnung der fachärztlichen Unterversorgung</u>

Fachärztlicher Versorgungsbedarf liegt definitorisch vor, wenn Empfehlungen oder Leitlinien einen regelmäßigen fachärztlichen Kontakt empfehlen oder wenn ein subjektives oder objektives, behandelbares und behandlungsbedürftiges Gesundheitsproblem besteht. In den Versorgungsbereichen, in denen keine gängigen Empfehlungen oder Leitlinien vorlagen, wurden die fachärztlichen Kontakte und die ärztliche Fallbewertung einbezogen. Das Konzept des fachärztlichen Versorgungsbedarfs wurde nach den ärztlichen Fallbewertungen festgelegt; als Indikator für ein bestehendes Gesundheitsproblem wurden das ärztliche Urteil und die fachärztlichen Kontakte herangezogen. Somit lag Versorgungsbedarf in den Versorgungsbereichen wie folgt vor:

Für den Versorgungsbereich Mundgesundheit besteht bei allen Bewohnern ein Versorgungsbedarf. Denn eine jährliche zahnärztliche Kontrolluntersuchung wird für die Gesamtbevölkerung zur Vorbeugung von Mund- und Zahnerkrankungen empfohlen [20]. Bei den Versorgungsbereichen Sehfähigkeit und Hörfähigkeit wurde nicht pauschal für jeden Bewohner ein Versorgungsbedarf angenommen, denn wenn Einschränkungen in der Seh- und Hörfähigkeit durch entsprechende Hilfsmittel (Brille und Hörgeräte) ausgeglichen werden, ist in diesen Versorgungsbereichen keine jährliche fachärztliche Kontrolluntersuchung notwendig. Daher wurde Versorgungsbedarf nur bei denjenigen Bewohnern angenommen, die gemäß ärztlicher Beurteilung als unterversorgt gelten und/oder die mindestens einen fachärztlichen Kontakt in den vorangegangenen 12 Monaten hatten. Beim Versorgungsbereich Parkinson-Syndrom fußt die Definition des Versorgungsbedarfs auf bestehenden Leitlinien, die mindestens einen jährlichen Kontakt zu Neurologen/Psychiatern empfehlen [21]. Somit wurde für alle Bewohner mit Parkinson-Diagnose ein neurologischer Versorgungsbedarf angenommen.

Fachärztliche Unterversorgung wurde berechnet, indem die Zahl der Bewohner mit fachärztlicher Unterversorgung durch die Zahl der Bewohner mit Versorgungsbedarf geteilt wurde. Außerdem wurde der Anteil der Bewohner berechnet, die in mindestens einem Versorgungsbereich unterversorgt waren. Bewohner, deren Versorgungssituation nicht beurteilbar war oder die als überversorgt galten, wurden von dieser Berechnung ausgeschlossen, da bei ihnen nicht eindeutig ermittelt werden konnte, ob sie einen Versorgungsbedarf aufwiesen oder nicht. Für den Anteil der fachärztlich unterversorgten Bewohner in der Stichprobe wurde jeweils das 95%-Konfidenzintervall berechnet.

<u>Non-Responder-Analyse und Hochrechnung</u>

Im Rahmen einer Non-Responder-Analyse wurde untersucht, ob sich die teilnehmenden Bewohner von den Heimbewohnern in Bremen und Niedersachsen unterscheiden, die nicht

teilgenommen haben. Hierfür wurden Routinedaten der AOK Niedersachsen und der AOK Bremen/Bremerhaven genutzt und mittels logistischer Regression mit Schätzung robuster Standardfehler ausgewertet. Die abhängige Variable war die Teilnahme an der Datenerhebung. Folgende unabhängigen Variablen wurden ins Modell aufgenommen: Alter, Geschlecht, Pflegegrad, Mortalität in den Jahren der Erhebung, Anzahl der Krankenhausaufenthalte, Anzahl der Hausarztkontakte, und diagnostizierte Erkrankungen.

Signifikante Zusammenhänge zwischen diesen soziodemografischen und gesundheitsbezogenen Merkmalen und der Teilnahmewahrscheinlichkeit geben Hinweise darauf, dass Unterschiede in der Teilnahmewahrscheinlichkeit bestehen. Diese Unterschiede in der Teilnahmewahrscheinlichkeit lassen sich bei der nachfolgenden Hochrechnung berücksichtigen. Es wurden zwei Hochrechnungen vorgenommen: Von der Stichprobe auf alle gesetzlich und privat versicherten Heimbewohner in (1) Bremen und Niedersachsen und (2) Deutschland. Als Datenbasis für die Hochrechnung wurde die Pflegestatistik zum Stichtag 31.12.2017 verwendet [22]. Sämtliche Analysen erfolgten mit den Softwareprogrammen R und Microsoft Excel.
