# Supplementary material for: Medical specialist undertreatment in nursing home residents—Prevalence and extrapolation
Source: Z Gerontol Geriatr. 2021 Mar 16;54(5):479–84. [Article in German] doi: 10.1007/s00391-021-01865-z (PMC8354900; doi:10.1007/s00391-021-01865-z)
Supplement: Supplementary file 2 [file 391_2021_1865_MOESM2_ESM.pdf]

Tabelle 1: Deskriptivstatistik der Stichprobe - Heimbewohner

		n	Anteil
**Geschlecht**	männlich	136	33 %
	weiblich	273	67 %
**Alter**	60-64	17	4 %
	65-69	34	8 %
	70-74	27	7 %
	75-79	54	13 %
	80-84	88	22 %
	85-89	79	19 %
	90+	110	26 %
**Pflegegrad**	1	2	0 %
	2	77	19 %
	3	154	38 %
	4	121	30 %
	5	49	12 %
	fehlende Daten	6	1 %
**analysierte Stichprobe**		**409**	**100 %**
