# Supplementary material for: Medical specialist undertreatment in nursing home residents—Prevalence and extrapolation
Source: Z Gerontol Geriatr. 2021 Mar 16;54(5):479–84. [Article in German] doi: 10.1007/s00391-021-01865-z (PMC8354900; doi:10.1007/s00391-021-01865-z)
Supplement: Supplementary file 3 [file 391_2021_1865_MOESM3_ESM.pdf]

Tabelle 2: Deskriptivstatistiken der Stichprobe - Heime

		n	Anteil
**Trägerart**	freigemeinnützig	21	49%
	privat	18	42%
	öffentlich	2	5%
	fehlende Angaben	3	5%
**Bundesland**	Bremen	24	56%
	Niedersachsen	16	37%
	fehlende Angaben	4	7%
**Einrichtungsplätze**	< 35 Plätze	4	9%
	36-69 Plätze	17	40%
	70-99 Plätze	12	28%
	> 100 Plätze	7	16%
	fehlende Angaben	4	7%
**analysierte Stichprobe**		**44**	**100 %**
