# Supplementary material for: Medical specialist undertreatment in nursing home residents—Prevalence and extrapolation
Source: Z Gerontol Geriatr. 2021 Mar 16;54(5):479–84. [Article in German] doi: 10.1007/s00391-021-01865-z (PMC8354900; doi:10.1007/s00391-021-01865-z)
Supplement: Supplementary file 4 [file 391_2021_1865_MOESM4_ESM.pdf]

Tabelle 3: Objektiver und subjektiver Gesundheitszustand der Pflegeheimbewohner nach Versorgungsbereich

**Versorgungsbereich**	**Erhebungsart**	**Merkmale**	**gültige Werte**	**Ø (Standardabweichung) bzw. % („trifft zu“)**
**Hörfähigkeit**	Assessment	Hörtest nicht bestanden	229	41 %
	Befragung Pflegebedürftiger (Selbsteinschätzung)	besitzt aktuell Hörgerät(e)	401	16 %
		trägt Hörgerät(e) aktuell nicht	61	34%
		Zufriedenheit mit Hörfähigkeit (in Schulnoten)	254	2,4 (1,1)
		unzufrieden mit Hörfähigkeit (Schulnote 5-6)	254	6 %
		Bewertung der Hörfähigkeit (in Schulnoten)	253	2,7 (1,1)
		negative Bewertung der Hörfähigkeit (Schulnote 5-6)	253	7 %
	Befragung Bezugspflegekraft (Fremdeinschätzung)	Bewohner kann nicht telefonieren	337	11 %
	Auswertung Pflegedokumentation	Diagnostizierte Erkrankungen des Hörapparats	29	7 %
**Sehfähigkeit**	Assessment	Sehtest Nähe UND Ferne binokular bestanden (=Visus-Wert > 0,3)	224	52 %
		Sehtest Nähe ODER Ferne binokular nicht bestanden (=Visus-Wert ≤ 0,3)	224	48 %
		Sehtest Fernsicht links ODER rechts nicht bestanden (=Visus-Wert ≤ 0,3)	213	52 %
	Befragung Pflegebedürftiger (Selbsteinschätzung)	besitzt aktuell eine Brille	402	67 %
		trägt Brille aktuell nicht	236	52%
		Zufriedenheit mit Sehfähigkeit (in Schulnoten)	257	2,7 (1,2)
		unzufrieden mit Sehfähigkeit (Schulnote 5-6)	257	10 %
		Bewertung der Sehfähigkeit (in Schulnoten)	256	2,7 (1,3)
		negative Bewertung der Sehfähigkeit (Schulnote 5-6)	256	11 %
	Befragung Bezugspflegekraft (Fremdeinschätzung)	Fremdeinschätzung: kann Kleingedrucktes nicht erkennen	320	23 %
	Auswertung Pflegedokumentation	Diagnostizierte Erkrankungen des Auges	72	18 %
**Mundgesundheit**	Assessment	OHAT Punktwert	256	1,4 (1,9)
		OHAT auffällig (mind. 1 Item mit Wert ≥ 1)	256	55 %
	Befragung Pflegebedürftiger (Selbsteinschätzung)	OHIP G-14 Punktwert	258	2,2 (5,2)
		OHIP G-14 auffällig (mind. 1 Item mit Score ≥ 2)	258	28 %
		aktuell Beschwerden im Mund/oder beim Essen	262	11%
		aktuell Schwierigkeiten beim Kauen	261	15%
	Befragung Bezugspflegekraft (Fremdeinschätzung)	weder natürliche Zähne noch Zahnprothesen	391	4 %
		besitzt Prothesen, trägt sie aber nicht	281	6%
**Parkinson-Syndrom**	Auswertung Pflegedokumentation	Diagnose Parkinson-Syndrom	31	8 %

Fortsetzung				
**Versorgungsbereich**	**Erhebungsart**	**Merkmale**	**gültige Werte**	**Ø (Standardabweichung) bzw. % („trifft zu“)**
**Allgemeinzustand**	Assessment	Mini-Mental-Status-Test (MMST)	276	19,2 (7,5)
		Mini-Mental-Status-Test (MMST) >24 (normale kognitive Funktion)	276	36%
		Cornell Scale for Depression in Dementia (CSDD)	402	29,7 (153,6)
		Cornell Scale for Depression in Dementia (CSDD) Score > 12 (Depressivität wahrscheinlich)	402	3%
	Befragung Pflegebedürftiger (Selbsteinschätzung)	Geriatrische Depressions-Skala (GDS-15)	259	3,4 (3,0)
	Befragung Bezugspflegekraft (Fremdeinschätzung)	Erweiterter Barthel-Index (EBI)	409	35,6 (17,2)

Anmerkung: Die Größe der analysierten Stichprobe betrug über alle Versorgungsbereiche hinweg 409.
