# Supplementary material for: Medical specialist undertreatment in nursing home residents—Prevalence and extrapolation
Source: Z Gerontol Geriatr. 2021 Mar 16;54(5):479–84. [Article in German] doi: 10.1007/s00391-021-01865-z (PMC8354900; doi:10.1007/s00391-021-01865-z)
Supplement: Supplementary file 5 [file 391_2021_1865_MOESM5_ESM.pdf]

Tabelle 4: (Fach)Ärztliche Versorgung von Pflegeheimbewohnern in den vergangenen 12 Monaten vor der Datenerhebung

	**Medizinische Fachgebiete und Versorgungsbereiche**				
	**Hausärztliche Versorgung (n=409)**	**Augenärztliche Versorgung (n=409)**	**HNO-ärztliche Versorgung (n=409)**	**Zahnärztliche Versorgung (n=409)**	**Neurologische/ Psychiatrische Versorgung (n=31)**
Bewohner mit mind. 1 Kontakt[1] (%)	400 (98 %)	69 (17 %)	88 (22 %)	133 (33 %)	6 (19 %)
Ärztliche Kontakte[1] pro Bewohner[2]					
Arithmetischer Mittelwert (Ø)	12,2	0,4	0,4	0,7	3,3
Standardabweichung	6,0	0,6	0,5	0,7	1,7
Spannweite (min; max)	0; 63	0; 11	0; 7	0; 9	0; 9
Bewohner mit mind. 1 Telefonkontakt (%)	305 (75 %)	14 (3 %)	11 (3 %)	21 (5 %)	15 (48 %)
Telefonische Kontakte/Bewohner[2]					
Arithmetischer Mittelwert (Ø)	4,8	0,1	0,0	0,1	0,1
Standardabweichung	6,4	0,3	0,3	0,4	1,1
Spannweite (min; max)	0; 52	0; 3	0; 3	0; 3	0; 5
Bewohner mit mind. 1 Pflegeheimvisite (%)	383 (94 %)	14 (3 %)	60 (15 %)	88 (22 %)	19 (61 %)
Visiten im Pflegeheim pro Bewohner[2]					
Arithmetischer Mittelwert (Ø)	6,9	0,1	0,2	0,3	0,1
Standardabweichung	6,5	0,4	0,6	0,8	2,1
Spannweite (min; max)	0; 62	0; 4	0; 3	0; 9	0; 7
Bewohner mit mind. 1 Praxiskontakt (%)	77 (19 %)	56 (14 %)	29 (7 %)	63 (15 %)	4 (13 %)
Praxiskontakte pro Bewohner[2]					
Arithmetischer Mittelwert (Ø)	0,5	0,3	0,1	0,3	0,0
Standardabweichung	1,8	0,9	0,7	0,9	1,4
Spannweite (min; max)	0; 23	0; 9	0; 7	0; 7	0; 6

[1] Telefonischer Kontakt, Visite im Pflegeheim oder Besuch in der ärztlichen Praxis.

[2] Bei Parkinson beschränkt auf diejenigen Bewohner, die eine Parkinsondiagnose aufweisen (n=31)
