# Supplementary material for: Medical specialist undertreatment in nursing home residents—Prevalence and extrapolation
Source: Z Gerontol Geriatr. 2021 Mar 16;54(5):479–84. [Article in German] doi: 10.1007/s00391-021-01865-z (PMC8354900; doi:10.1007/s00391-021-01865-z)
Supplement: Supplementary file 6 [file 391_2021_1865_MOESM6_ESM.pdf]

Tabelle 5: Ärztliche Fallbewertungen

Versorgungsbereich	analysierte Stichprobe	eingeschlossene Fälle	bewertbare Fälle			nicht bewertbare Fälle
			bedarfsgerecht versorgt	unterversorgt	überversorgt	
**Sehfähigkeit**	409	409	348	52	3	6
**Hörfähigkeit**	409	409	385	15	1	8
**Mundgesundheit**	409	409	337	64	1	7
**Parkinson-Syndrom**	409	31	25	6	-	-
