# Supplementary material for: Medical specialist undertreatment in nursing home residents—Prevalence and extrapolation
Source: Z Gerontol Geriatr. 2021 Mar 16;54(5):479–84. [Article in German] doi: 10.1007/s00391-021-01865-z (PMC8354900; doi:10.1007/s00391-021-01865-z)
Supplement: Supplementary file 7 [file 391_2021_1865_MOESM7_ESM.pdf]

Tabelle 6: Non-Responder-Analyse

	Koeffizient	Standardfehler	p-Wert
Alter in Jahren	-0,030	0,010	0,000
Mann (Referenzkategorie: Frau)	0,140	0,170	0,410
Pflegegrad 3 (Referenzkategorie: Pflegegrad 2)	0,260	0,240	0,291
Pflegegrad 4 (Referenzkategorie: Pflegegrad 2)	0,310	0,240	0,203
Pflegegrad 5 (Referenzkategorie: Pflegegrad 2)	-0,290	0,290	0,323
Tod	-0,710	0,300	0,020
Anzahl an Krankenhausfälle	0,080	0,060	0,209
Anzahl Hausarztfälle	-0,150	0,040	0,000
Eine Erkrankungsdiagnose	10,496	1,060	0,000
Mind. 2 Erkrankungsdiagnosen	10,345	0,370	0,000
Konstante	-12,530	0,770	0,000
N	27,276		
McFadden R²	0,021		
AIC	2170,920		

Anmerkung: Logistische Regression mit robusten Standardfehlern; abhängige Variable: Teilnahme an der Datenerhebung: ja/nein, Datenquelle: Datenerhebung und Routinedaten der AOK Bremen/Bremerhaven und AOK Niedersachsen aus dem Jahr 2018.
